# Comparison of three routine methods for the identification of *Kerstersia gyiorum* from routine diagnostics and review of the literature

**DOI:** 10.3389/fmicb.2026.1901541

**Published:** 2026-10-05

**Authors:** Lara Purschwitz, Boyke Bunk, Percy Schröttner

**Affiliations:** 1 Institute for Clinical Chemistry and Laboratory Medicine, Faculty of Medicine and University Hospital Carl Gustav Carus, TUD Dresden University of Technology, Dresden, Germany; 2 Leibniz Institute DSMZ-German Collection of Microorganisms and Cell Cultures GmbH, Braunschweig, Germany; 3 Institute for Medical Microbiology and Virology, Faculty of Medicine and University Hospital Carl Gustav Carus, TUD Dresden University of Technology, Dresden, Germany

**Keywords:** dDDH, *Kerstersia gyiorum*, MALDI TOF MS, NGS, sequencing of the 16S rRNA gene, VITEK 2

## Abstract

**Introduction:**

Since its first description in 2003, clinical case reports have repeatedly documented that the bacterial species *Kerstersia gyiorum* is a human pathogen. *K. gyiorum* has been described in the context of wound and middle ear infections, has been found in urine samples, and has even been recognized as a causative agent of septicemia. However, the pathogenic potential of *K. gyiorum* cannot be clearly determined due to the current lack of information about this species. To reveal the clinical significance of *K. gyiorum*, it is therefore essential to first create a clearly defined reference collection of isolates obtained from routine clinical diagnostics.

**Methods:**

The aim of this study is to determine the most appropriate method for identifying *K. gyiorum* in routine diagnostics. To this end, three widely used routine methods (VITEK 2, MALDI-TOF MS, and 16S rRNA gene sequencing) were employed, and the accuracy of their results for *K. gyiorum* identification was compared. To ensure accurate species identification, dDDH (an NGS-based identification strategy) was used as the reference method. Furthermore, this manuscript provides an overview of all cases reported in the literature to date and discusses the accuracy of the identification results reported in these studies. In addition, genomic studies (pangenome and ANI matrix) were conducted using in silico analyses to investigate the clonal relationships among the isolates.

**Results:**

The results show that MALDI-TOF MS is best suited for identifying *K. gyiorum* in routine diagnostics. However, in contrast to most publications, 16S rRNA gene sequencing was not suitable, as it was impossible to distinguish *K. gyiorum* from other members of the Alcaligenaceae family. The VITEK 2 system must also be considered unreliable for the correct identification of *K. gyiorum*. Additionally, this study identified a candidate for an undescribed species of the genus Kerstersia.

**Discussion:**

MALDI-TOF MS is well suited for expanding the reference collection directly from routine diagnostics. The results of 16S gene sequencing should be interpreted with caution. Conversely, biochemical methods must be regarded as unreliable. The species’ large core genome suggests a specialized previously unknown specialized ecological niche. Furthermore, two distinct clusters within the reference collection suggest a common origin. Additionally, follow-up studies must determine whether one isolate that cannot be unambiguously assigned to a species represents a previously undescribed member of the genus Kerstersia.

## Introduction

*Kerstersia gyiorum* is a Gram-negative proteobacterium of the class *Betaproteobacteria* belonging to the *Alcaligenaceae* family. Based on the initial description, it grows as a solitary, paired, or short-chain form under aerobic conditions. The type strain (CIP 108214^T^) was isolated from a human ankle wound (Coenye et al., 2003). Furthermore, the colonies were white to grayish in color, with irregularly spreading edges and a sponge-like appearance (Coenye et al., 2003; Pence et al., 2013). Additionally, growth was detected at NaCl concentrations between 0 and 4.5% and at temperatures between 28 and 40 °C (Coenye et al., 2003). *K. gyiorum* exhibits negative oxidase, glucose, and urease reactions but a positive catalase reaction. These properties distinguish it phenotypically from other members of the *Alcaligenaceae* family. Furthermore, *K. gyiorum* lacks the fruity odor typical of members of the *Alcaligenaceae* family (Coenye et al., 2003; Pence et al., 2013; Almuzara et al., 2012; Baran et al., 2017). In addition, *K. gyiorum* was detected in the intestines of the housefly *Musca domestica,* from the blowhole of Yangtze finless porpoises (*Neophocaena asiaeorientalis asiaeorientalis*) living in captivity and in healthy free-living brown-throated sloths (*Bradypus* var*iegatus*) (Almuzara et al., 2012; Gupta et al., 2012; Wan et al., 2016; Carhuaricra-Huaman et al., 2024). Figure 1 summarizes all information where an infection with *K. gyiorum* has been reported in humans to date.

**Figure 1:**
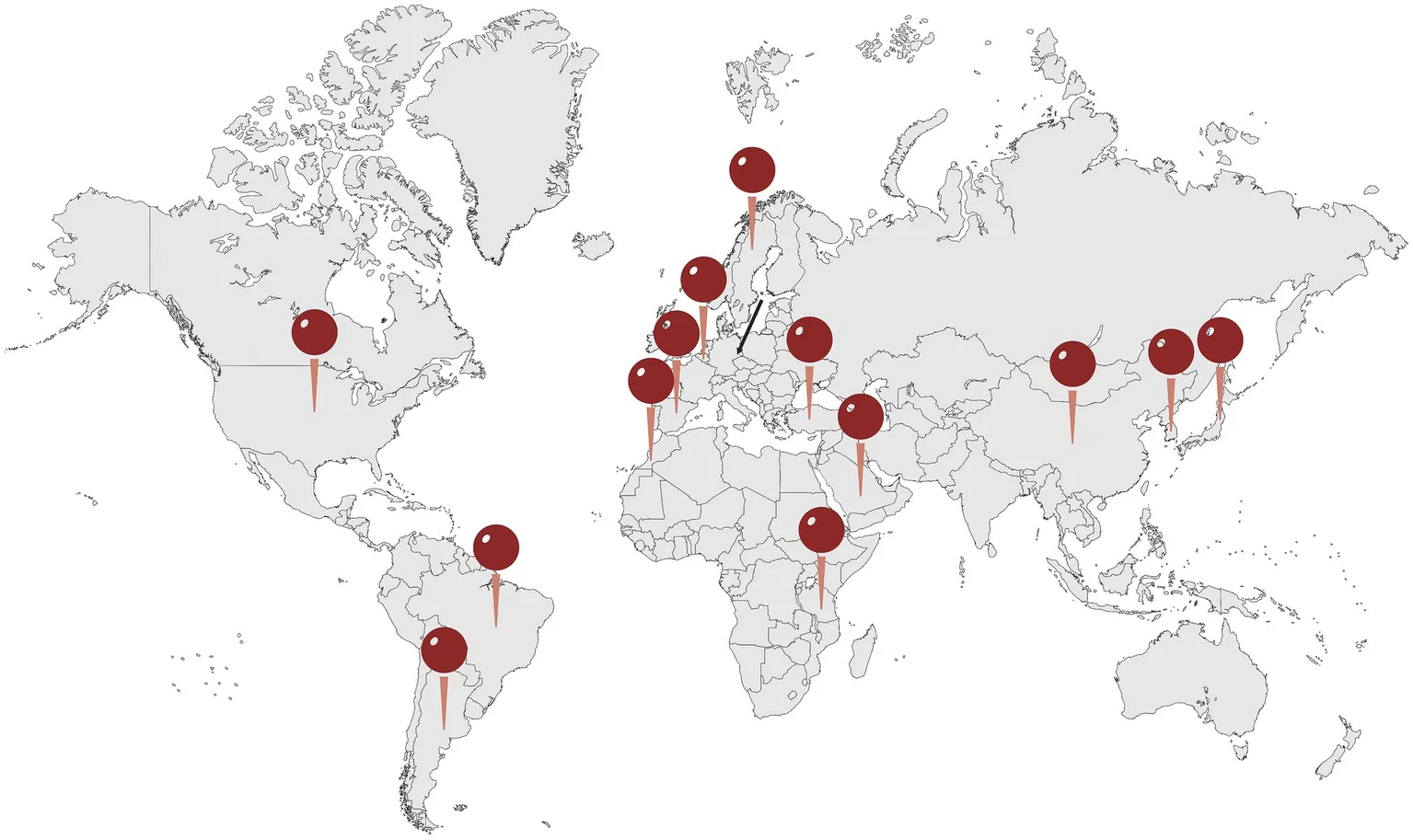
Global distribution of cases reporting on infections caused by *Kerstersia gyiorum* (red needle). Clinical collection UKD (black arrow). The image was generated using BioRender.

The genus *Kerstersia* includes a second species, *Kerstersia similis*. The type strain of this species (LMG 5890^T^, CCUG 46999^T^) originates from leg wounds as well. It was first described in 2012 (Vandamme et al., 2012).

However, as there are currently only very few case reports available, the clinical significance of the species cannot yet be reliably assessed. Therefore, to elucidate its pathogenicity in humans, a prospective, systematic approach is needed. The first step in this endeavor, and an absolute prerequisite, is to establish a well-characterized collection of *K. gyiorum* isolates. As the isolates used for such an investigation originate from routine clinical diagnostics, it is important to know if there is an identification strategy that reliably identifies *K. gyiorum*. This knowledge is relevant to continuously expanding the reference collection over time.

In most previous publications, identification was achieved by sequencing the 16S rRNA gene and MALDI-TOF MS (Coenye et al., 2003; Mwalutende et al., 2014). The results of MALDI-TOF MS analysis (the most used was that of Bruker Daltonik GmbH, Bremen, Germany) and sequencing of the 16S rRNA gene have consistently led to reliable identification of the organism (Coenye et al., 2003; Pence et al., 2013; Özcan, 2018). Furthermore, it has been reported that species-level identification using VITEK 2 is not possible because the profile of *K. gyiorum* is not yet included in the VITEK 2 database (Pence et al., 2013; Almuzara et al., 2012; Mwalutende et al., 2014; Özcan, 2018). However, a major limitation of these studies was that the reference methods used therein are questionable in terms of the accuracy of the identification results for *K. gyiorum*. Nowadays, methods based on whole-genome analysis, such as digital DNA–DNA hybridization (dDDH) or Average Nucleotide Identity (ANI), are considered the gold standard for species identification (Goris et al., 2007; Rosselló-Móra et al., 2011). Given that dDDH boasts a high level of aptitude for conducting taxonomic comparative analyses, the method was selected as the reference (Auch et al., 2010). By clarifying which routine method is best suited for species identification, we also hope to contribute to a better understanding of the frequency of *K. gyiorum* in clinical contexts in the future.

## Materials and methods

### Comprehensive literature search

A systematic literature search was conducted in PubMed and Google Scholar using the search term “*Kerstersia gyiorum*.” The search period covered the years 2003 through June 2026. Given the objective of comprehensively documenting the extant literature, no explicit inclusion or exclusion criteria were stipulated. The results of the literature search are summarized in Tables 1, 2.

**Table 1:** Overview of previously described infections caused by *Kerstersia gyiorum*.

Patient: age/sex	Location	Main diagnosis	Comorbidities	Social factors	Outcome	Reference
85/F	Gothenburg, Sweden	N/A	N/A	N/A	N/A	Coenye et al. (2003)
N/A	N/A	N/A	N/A	N/A	N/A	Coenye et al. (2003)
N/A	N/A	N/A	N/A	N/A	N/A	Coenye et al. (2003)
N/A	N/A	N/A	N/A	N/A	N/A	Coenye et al. (2003)
N/A	N/A	N/A	N/A	N/A	N/A	Coenye et al. (2003)
N/A	Belgium	N/A	N/A	N/A	N/A	Coenye et al. (2003)
55, M	St. Louis, Missouri, USA	Bilateral otorrhea	Post-mastoidectomy on both sides	Alcohol abuse, nicotine abuse	Survived	Pence et al. (2013)
61, F	Iowa, USA	Left lower leg ulcer	Morbid obesity, a condition after phlegmon	N/A	Survived	Pence et al. (2013)
69, F	Durham, North Carolina, USA	Bacteremia and sepsis	3 ulcers on the right lower leg, dizziness, diarrhea, schizophrenia	Schizophrenia	Survived	Bostwick et al. (2015)
16, M	Buenos Aires, Argentina	Complicated cholesteatomatous chronic otitis media with peripheral facial paralysis	Post-otitis media in the left ear and retro auricular abscess	Overcrowded and unhygienic living conditions	Survived	Almuzara et al. (2012)
63, F	Ohio, USA	Dialysis infection, catheter site, fever, hypotension	Chronic tracheostomy due to respiratory insufficiency, renal insufficiency, post-stroke	N/A	Deceased	Deutscher et al. (2014)
25, M	Sivas, Turkey	Purulent otorrhea, perforation of the tympanic membrane on both sides	Chronic purulent otitis media, hearing loss	Unencumbered	Survived	Uysal et al. (2015)
21, F	Diyardakbir, Turkey	Otorrhea and hearing loss with perforation of the eardrum and cholesteatoma on the left	None	Unencumbered	Survived	Özcan (2018)
21, M	Diyardakbir, Turkey	Chronic purulent otorrhea on the right	None	Nicotine abuse	Survived	Özcan (2018)
27, M	Diyardakbir, Turkey	Bilateral otorrhea, perforation of the eardrum, cholesteatoma, hearing loss	None	Unencumbered	Survived	Özcan (2018)
53, M	Mwanza, Tanzania	Otorrhea on the left for 2.5 years, hearing loss	None	Alcohol abuse, nicotine abuse	Survived	Mwalutende et al. (2014)
33, M	Mwanza, Tanzania	Otorrhea on the right for 1.5 years, hearing loss	None	Unencumbered	Survived	Mwalutende et al. (2014)
47, M	Ankara, Turkey	Right foot and ankle ulcers	Buerger’s disease (thromboangiitis obliterans). Post toe amputation	Nicotine abuse, poor personal hygiene	Survived	Baran et al. (2017)
70, F	Luzhou, China	Respiratory tract infection	Tracheostoma and epilepsy	Unencumbered	Survived	Li et al. (2019)
82, M	Nara, Japan	Dysuria	Bladder cancer, removal of the urinary bladder, including chemotherapy and percutaneous nephrostomy	Unencumbered	Survived	Ogawa et al. (2016)
48, M	Nara, Japan	Bacterial superinfection of congestive dermatitis	Congestive dermatitis, athlete’s foot	Low awareness of illness and low compliance	Survived	Kitagawa et al. (2021)
47, M	Changsha, China	Gangrene of the left lower leg	Chronic osteomyelitis	Unencumbered	Survived	Lan et al. (2017)
30, F	Istanbul, Turkey	Pain, otorrhea, and eardrum perforation in the left ear	Chronic purulent otitis media on the left with hearing loss, dizziness and tinnitus	N/A	Survived	Borsa et al. (2017)
88, M	Zaragoza, Spain	Otalgia and otorrhea in the right ear	Diabetes mellitus type II, hypertension, dyslipidemia, chronic renal insufficiency, cardiac insufficiency, prostatic hypertrophy, post-perforation right eardrum, and otorrhea	Unencumbered	Survived	Vela et al. (2017)
51, F	Seoul, South Korea	Otorrhea left ear	Oost mastoidectomy on both sides	N/A	Survived	Kim et al. (2018)
13, F	Riyadh, Saudi-Arabien	Chronic perichondritis, ear discharge, gastroenteritis	Cerebral palsy, developmental delay, blindness, epilepsy, gastrostomy tube (puncture red and hot)	Unencumbered	N/A	AlSunbul et al. (2024)
92, F	Hangzhou, China	Impaired consciousness, pneumonia	Alzheimer’s disease, high blood pressure	Unencumbered	Survived	Sun et al. (2023)
78, M	Hangzhou, China	Coma, fever, purulent sputum	Parkinson’s disease, high blood pressure, tracheotomy	Unencumbered	Survived	Sun et al. (2023)
85, F	Rio de Janeiro, Brazil	Chronic wound on the right instep	Chronic venous insufficiency, diabetes mellitus	N/A	N/A	Pires et al. (2020)
69, F	Chengdu, China	Swelling, tenderness, extensive erythema, skin ulceration with purulent discharge, epidermal desquamation, and foul odor on the right leg	Varicose veins, renal insufficiency, hyperuricemia, malnutrition, anemia	Unencumbered	Survived	Qin et al. (2025)
52, F	Chifeng, China	Dizziness, fluid accumulation in the external ear canal for several years, and impaired hearing	Nothing described	Alcohol consumption in the past	Survived	Shen et al. (2025)
64, M	Yibin, China	Chronic osteomyelitis with persistent discharge and 2 ulcerations on the right lower limb	Right tibial shaft fracture more than 10 years ago, hypertension, suffered cerebral infarction with left-sided hemiplegia	Uncompliant	Survived	Zhang et al. (2025)
39, M	Shreveport, Louisiana, USA	Chronic suppurative otitis media, perforation of the right tympanic membrane	History of multiple tympanostomy tube placements during childhood	Nicotine abuse	Survived	Holmes et al. (2021)
73, M	Tangier, Marocco	Cranial injury after trauma, bilateral oto-mastoid fillings	Type 2 diabetes, arterial hypertension	N/A	Survived	El Bardouni et al. (2026)

N/A, not available; F, female; m, male.

**Table 2:** Overview of the samples and their locations, species identification, and the microbial spectrum as reported in the literature.

Patient: age/sex	Preanalytics (collection site and sample type)	Analysis (species identification)	Spectrum of pathogens (mono- or polymicrobial)	Reference
85, F	Stool	N/A	N/A	Coenye et al. (2003)
N/A	Swab leg wound	N/A	N/A	Coenye et al. (2003)
N/A	Swab leg wound	N/A	N/A	Coenye et al. (2003)
N/A	Swab leg wound	N/A	N/A	Coenye et al. (2003)
N/A	Swab ankle wound	N/A	N/A	Coenye et al. (2003)
N/A	Sputum	N/A	N/A	Coenye et al. (2003)
55, M	Left mastoid cavity	MALDI-TOF MS/16S rRNA gene sequencing	*Corynebacterium amycolatum, Gram-negative coccobacillus*	Pence et al. (2013)
61, F	Ulcer left lower leg	MALDI-TOF MS/16S rRNA gene sequencing	*Morganella morganii*	Pence et al. (2013)
69, F	(Aerobic) blood culture	16S rRNA gene sequencing	None	Bostwick et al. (2015)
16, M	Bezold’s abcess	16S rRNA gene sequencing, *gyrB* gene sequencing	N/A	Almuzara et al. (2012)
63, F	Bronchial alveolar lavage	MALDI TOF MS	*Pseudomonas aeruginosa*, *Stenotrophomonas maltophilia*	Deutscher et al. (2014)
25, M	Swab right ear drainage	MALDI-TOF MS (Becton Dickinson)	N/A	Uysal et al. (2015)
21, F	Swab left ear drainage	MALDI TOF MS	None	Özcan (2018)
21, M	Swab right ear drainage	MALDI TOF MS	None	Özcan (2018)
27, M	Abscess of the right middle ear	MALDI TOF MS	*Morganella morganii*	Özcan (2018)
53, M	Swab left ear drainage	MALDI TOF MS	*Proteus mirabilis*	Mwalutende et al. (2014)
33, M	Swab on the right ear drainage	MALDI TOF MS	*Staphylococcus aureus*, *Escherichia coli*	Mwalutende et al. (2014)
47, M	Wound swab ankle and dorsum of right foot	MALDI-TOF MS/16S rRNA gene sequencing	*Proteus vulgaris*, *Enterobacter cloacae*, *Morganella morganii*	Baran et al. (2017)
70, F	Sputum	MALDI-TOF MS/16S rRNA gene sequencing (whole genome sequencing)	N/A	Li et al. (2019)
82, M	Urine	MALDI-TOF MS (Bruker), 16S rRNA gene sequencing	*Proteus vulagaris*	Ogawa et al. (2016)
48, M	Swab leg wound left	16S rRNA gene sequencing	*Bordetella trematum, Alcaligenes faecalis, Corynebacterium* spp.	Kitagawa et al. (2021)
47, M	Wound swab gangrene left leg	MALDI-TOF MS/16S rRNA gene sequencing	*Acinetobacter baumanii*	Lan et al. (2017)
30, F	Swab left ear drainage	MALDI-TOF MS/16S rRNA gene sequencing	N/A	Borsa et al. (2017)
88, M	Swab right ear drainage	MALDI-TOF MS	*Staphylococcus aureus*	Vela et al. (2017)
51, F	Swab left ear discharge	MALDI-TOF MS/16S rRNA gene sequencing	Diphteroid^a^	Kim et al. (2018)
13, F	Swab right ear	MicroScan WalkAway Plus, MALDI-TOF MS	N/A	AlSunbul et al. (2024)
92, F	Sputum	MALDI-TOF MS/16S rRNA gene sequencing	Mixed infection	Sun et al. (2023)
78, M	Sputum	MALDI-TOF MS/16S rRNA gene sequencing	Mixed infection	Sun et al. (2023)
85, F	Swab wound right foot	MALDI-TOF MS/16S rRNA gene sequencing	*Staphylococcus aureus*, *Pseudomonas aeruginosa*	Pires et al. (2020)
69, F	Wound exudate right leg	MALDI-TOF MS/16S rRNA gene sequencing	*Proteus mirabilis*, *Pseudomonas aeruginosa*, *Providencia stuartii*	Qin et al. (2025)
52, F	Swab right ear	Whole Genome Sequencing	*Alcaligenes faecalis* subsp. faecalis	Shen et al. (2025)
64, M	Swab debridement right leg	MALDI-TOF MS/16S rRNA gene sequencing	N/A	Zhang et al. (2025)
39, M	Fluid oft the right external auditory canal	N/A	N/A	Holmes et al. (2021)
73, M	Swab ear discharge bilateral	MALDI-TOF MS/16S rRNA gene sequencing	*Escherichia coli*, *Achromobacter xylosoxidans*	El Bardouni et al. (2026)

a Taken from the publication, no further details were provided.

### Establishing a clinical collection of *Kerstersia gyiorum* from routine diagnostics

Over 8 years, a total of 15 strains, primarily identified as *K. gyiorum,* were collected during routine diagnostics at the Institute of Medical Microbiology and Virology of the University Hospital Dresden, Germany (see details in Table 3).

**Table 3:** Clinical information connected to the strains of the collection of *Kerstersia gyiorum* from Dresden over the period December 2012–July 2020.

DSM No	Sex	Age	source	Detected microbial spectrum	Remarks/problems
103762	M	61	Throat swab	Oral microflora, *Neisseria* spp., *Klebsiella pneumoniae*, *Candida albicans*	Diabetic foot syndrome (DFS)
105837	M	51	Ear canal swab	Skin flora, *Escherichia coli*	Cholesteatome, hearing loss due to sound perception and conduction disorder
107564	M	22	Ear canal swab	Skin flora, *Proteus mirabilis*	Chronic inflammation of the middle ear
107745	F	43	Wound swab lower leg	Skin flora, *Pseudomonas aeruginosa*, *Providentia stuartii*	DFS
108176	M	74	Urine (from catheter)	*Proteus mirabilis, Providentia rettgeri*	Urinary tract infection, multiple sclerosis, acute respiratory insufficiency, anemia, hypokalemia
108180	F	77	Wound swab lower leg	*Serratia marcescens*, *Morganella morganii*, *Proteus vulgaris*, anaerobeskin flora	Femoral neck fracture, decubitus grade 2 (ischium), bronchial asthma, hyperuricemia, hypertension, venous insufficiency
109001	F	66	Wound swab lower leg	*Myroides odoratimimus*, *Pseudomonas aeruginosa*	Leg ulcer
109002	F	52	Urine	Germs of the anterior urethra, *Escherichia coli*, *Morganella morganii*, *Klebsiella pneumoniae*	N/A
109003	F	77	Wound swab lower leg	Skin flora, *Staphylococcus aureus*, *Pseudomonas aeruginosa*, *Providencia rettgeri*, coliform bacterium (no further differentiation possible)	DFS, wound healing disorder, varices of the lower extremity
109564	F	67	Wound swab lower leg	Skin flora, *Serratia marcescens*, *Proteus vulgaris/hauseri*, *Pseudomonas aeruginosa*, haemolytic streptococci group C	Leg ulcer, alimentary zinc deficiency, hyperuricemia, hypertension, varicose veins
110458	F	80	Wound swab lower leg	Skin flora, anaerobe skin flora, *Streptococcus pyogenes*, *M. morganii*, *Pseudomonas aeruginosa*, *Bacteroides fragilis*	Leg ulcer
110459	F	57	Wound swab wound healing disorder	*Morganella morganii*, *Pseudomonas aeruginosa*, *Proteus vulgaris*, *Citrobacter koseri*, *Enterobacter faecalis*, *Prevotella bivia*, *Anaerococcus* spp.*, Peptoniphilus harei*	Intraoperative smear, wound healing disorder after surgery, right inguinal
111616	M	84	Urine (from catheter)	Germs of the anterior urethra, *Escherichia coli, Pseudomonas aeruginosa*	Suspected urinary tract infection
111617	M	83	Wound swab lower leg	*Pseudomonas aeruginosa*, *Providencia stuartii*, *Proteus mirabilis*, *Streptococcus dysgalactiae*	Leg ulcer, vitamin B12 deficiency, alimentary zinc deficiency, acute bleeding anemia, hypertension, decubitus grades 1 and 2 heel, chronic kidney disease stage 2
111618	F	80	Wound swab	Skin flora, *P. mirabilis, P. aeruginosa,* haemolytic streptococci group C	N/A

DFS, diabetic foot syndrome; N/A, not available.

All collected strains were subsequently stored in a Mikrobank™ conservation system (Pro-Lab Diagnostics™, Fisher Scientific, Schwerte, Germany). Furthermore, these strains were additionally deposited in the “Open Collection” of the Leibniz Institute DSMZ-German Collection of Microorganisms and Cell Cultures GmbH (Braunschweig). In addition, the type strain of the species *K. gyiorum* (DSM 16618^T^) was purchased from the DSMZ collection and used as a reference for this study. Consequently, a total of 16 strains, including the type strain and 15 derived from routine diagnostics, were examined in the present study. The 15 isolates in this study were obtained from the institute’s routine diagnostic testing and are herein referred to as the “clinical collection.”

### Whole genome analysis of *Kerstersia gyiorum* and establishing a reference collection

Whole-genome analysis was conducted using the PacBio platform. Genomic DNA extraction was carried out using MasterPure™ Gram Positive DNA Purification Kits from Epicentre®Biotechnologies Germany according to the manufacturer’s instructions. SMRTbell™ template library was prepared according to the instructions from Pacific Biosciences, Menlo Park, CA, USA, following the Procedure & Checklist—Preparing Multiplexed Microbial Libraries Using SMRTbell® Express Template Prep Kit 2.0. Briefly, for preparation of 10 kb libraries, 1 μg genomic DNA was shared using the Megaruptor 3 from Diagenode, Denville, NJ, USA, according to the manufacturer’s instructions. DNA was end-repaired and ligated to barcoded adapters using components from the SMRTbell Express Template Prep Kit 2.0 from Pacific Biosciences, Menlo Park, CA, USA. Reactions were carried out according to the manufacturer’s instructions. Samples were pooled according to the calculations provided by the Microbial Multiplexing Calculator. Conditions for annealing of sequencing primers and binding of polymerase to purified SMRTbell™ template were assessed with the Calculator in SMRT®link, Pacific Biosciences, Menlo Park, CA, USA. Libraries were sequenced on the Sequel *IIe* (Pacific Biosciences, Menlo Park, CA, USA) taking one 15 h movie per SMRT cell.

Long read genome assembly was performed with the “Microbial Assembly” protocol included in SMRTlink version 10.1. In all of the cases, one circular bacterial chromosome of 4 Mbp was obtained with a coverage of 100x. The bacterial chromosome was rotated to the *dnaA* gene afterwards. Smaller circular contigs were designated as plasmids and rotated to their replication genes. No other contigs or contaminations were obtained or reported (complete genome sequencing).

Finally, genome sequences of the isolates were deposited within NCBI GenBank. GenBank Accession numbers are provided in Table 4. Genome annotation was done based on the Prokaryotic Genome Annotation Pipeline (PGAP) (Tatusova et al., 2016).

**Table 4:** Comparison of the identification of *Kerstersia gyiorum* using routine microbiological methods and the dDDH as a reference method.

DSM number	MALDI TOF MS	VITEK 2	Sequencing of the 16S-rRNA gene	Top hit percent identity	16S seq length	GenBank Accession No.	Genome-based ID (dDDH)
16618^T^	*Kerstersia gyiorum* (2.33)	No identification possible	*Kerstersia gyiorum*: 100%	Kerstersia gyiorum: 100%	876	JBXEKF000000000	*Kerstersia gyiorum* (100%)
			*Bordetella* sp. 100%				
103762	*Kerstersia gyiorum* (2.27)	Pseudomonas fluorescens	*Kerstersia gyiorum*: 99.36%	*Kerstersia gyiorum*: 99.86%	948	JBXEKT000000000	*K. gyiorum* (90.1%)
			*Bordetella* sp.: 99.86%				
105837	*Kerstersia gyiorum* (1.94)	*Pandoraea* spp. (93%)	*Kerstersia gyiorum*: 99.88%	Kerstersia gyiorum: 100%	1183	JBXEKS000000000	“Potentially new species” (29.4%)
			*Bordetella* sp. 99.88%				
			*Alcaligines* sp. 100%				
107564	*Kerstersia gyiorum* (2.24)	*Pseudomonas fluorescens*	*Kerstersia gyiorum*: 99.86%	*Kerstersia gyiorum*: 99.86%	1151	JBXEKR000000000	*Kerstersia gyiorum* (90.1%)
			*Bordetella* sp.: 99.36%				
107745	*Kerstersia gyiorum* (2.30)	No identification possible	*Kerstersia gyiorum*: 99.78%	*Bordetella* sp. 99.78%	1093	JBXEKQ000000000	*Kerstersia gyiorum* (91,5%)
			*Bordetella* sp. 99.78%				
108176	*Kerstersia gyiorum* (2.27)	*Pseudomonas alcaligenes*	*Kerstersia gyiorum*: 99.83%	*Kerstersia gyiorum*: 99.83%	920	JBXEKP000000000	*Kerstersia gyiorum* (90.7%)
			*Bordetella* sp.: 99.83%				
108180	*Kerstersia gyiorum* (2.36)	No identification possible	*Kerstersia gyiorum*: 99.67%	*Bordetella* sp.: 99.67%	911	JBXEKO000000000	*Kerstersia gyiorum* (90,2%)
			*Bordetella* sp. 99.51%				
109001	*Kerstersia gyiorum* (2.26)	No identification possible	*Kerstersia gyiorum*: 99.86%	*Bordetella* sp.: 99.86%	1012	JBXEKN000000000	*Kerstersia gyiorum* (80.7%)
			*Bordetella* sp.: 99.86%				
109002	*Kerstersia gyiorum* (2.28)	*Pandoraea* spp. (99%)	*Kerstersia gyiorum*: 99.86%	Kerstersia gyiorum: 99.86%	1007	JBXEKM000000000	*Kerstersia gyiorum* (89%)
			*Bordetella* sp.: 99.79%				
109003	*Kerstersia gyiorum* (2.28)	No identification possible	*Kerstersia gyiorum*: 99.88%	*Kerstersia gyiorum*: 100%	817	JBXEKL000000000	*Kerstersia gyiorum* (90.2%)
			*Bordetella* sp.: 99.88%				
109564	*Kerstersia gyiorum* (2.28)	*Pandoraea* spp. (99%)	*Kerstersia gyiorum*: 99.89%	*Bordetella* sp. 99.89%	1136	JBXEKK000000000	*Kerstersia gyiorum* (81,2%)
			*Bordetella* sp.: 99.89%				
110458	*Kerstersia gyiorum* (2.25)	No identification possible	*Kerstersia gyiorum*: 99.73%	*Kerstersia gyiorum*: 99.73%	985	JBXEKJ000000000	*Kerstersia gyiorum* (81.5%)
			*Bordetella* sp.: 99.73%				
110459	*Kerstersia gyiorum* (2.41)	*Pseudomonas fluorescens*	*Kerstersia gyiorum*: 99.79%	Kerstersia gyiorum: 99.79%	1017	JBXEKI000000000	*Kerstersia gyiorum* (90,3%)
			*Bordetella* sp.: 99.68%				
111616	*Kerstersia gyiorum* (2.36)	*Pandoraea* spp. (97%)	*Kerstersia gyiorum*: 99.68%	*Kerstersia gyiorum*: 99.68%	1021	JBXEKH000000000	*Kerstersia gyiorum* (91.1%)
			*Bordetella* sp.: 99.68%				
111617	*Kerstersia gyiorum* (2.27)	No identification possible	*Kerstersia gyiorum*: 99.91%	Kerstersia gyiorum: 99.91%	1151	JBXEKG000000000	*Kerstersia gyiorum* (90.6%)
			*Bordetella* sp. 99.91%				
111618	*Kerstersia gyiorum* (2.25)	*Pseudomonas fluorescens*	*Kerstersia gyiorum*: 99.69%	*Kerstersia gyiorum*: 99.69%	1030	JBXEKF000000000	*Kerstersia gyiorum* (90.8%)
			*Bordetella* sp.: 99.58%				

Genome data obtained from whole-genome sequencing was then transferred to the Type Strain Genome Server (TYGS)^1^ in nucleotide FASTA format. This data was used for phylogenetic analysis and taxon delimitation, which can differentiate between species (Goris et al., 2007). It has been considered that a dDDH value of 70% or higher between two organisms is considered a reliable indicator for species identification (Goris et al., 2007; Auch et al., 2010). The dDDH was used both as the reference method for comparing different routine methods and to establish a reference collection for further research on the *K. gyiorum* species. All isolates that showed an identity of 70% or higher for dDDH were included in this so-called “reference collection.”

The online tool Genome-to-Genome-Distance-Calculator (GGDC), available at https://ggdc.dsmz.de/ggdc.php, was used for a more precise analysis between three genome pairs. This tool can indicate the genetic distance between two freely selectable genomes via *in silico* dDDH calculation (Auch et al., 2010; Meier-Kolthoff et al., 2013; Meier-Kolthoff et al., 2022). Although TYGS is more useful for more comprehensive analyses, both tools are equally reliable (Meier-Kolthoff et al., 2022). The information is summarized in Table 4.

To further investigate the possible clonal origin of individual strains, an ANI matrix was created using the online tool JSpeciesWS (Richter et al., 2016). The raw data are provided in Supplementary Table 1. The results were visualized using Morpheus (Hierarchical clustering and heatmap visualization software; Broad Institute, Cambridge, MA, USA). Furthermore, a pan-genome analysis and, subsequently, a Presence-Absence Visualization Analysis (PAV) were performed on the 14 isolates from the reference collection. The PanExplorer was utilized for this purpose (Dereeper et al., 2022).

### Identification of *Kerstersia gyiorum* using VITEK 2

First, the isolates from the cryotubes were recultivated. For this purpose, a 3-eyelet smear on Columbia blood agar containing 5% sheep blood (Oxoid, Wesel, Deutschland) was conducted. The bacteria were then incubated at 37 °C for 18–24 h, in accordance with the EUCAST guidelines (version 12.0, 01 January 2022) (EUCAST, 2022). Next, a bacterial suspension with a turbidity of 0.5 McFarland was prepared (EUCAST, 2022) in 2.5 mL of 0.45% NaCl (saline) solution, using the DensiCheck device (bioMérieux, Nürtingen, Germany) and a minishaker (IKA, Staufen, Germany).

The Vitek 2 system with a GN ID card was then used for bacterial identification, as previously described by our group (Schröttner et al., 2014). The results are summarized in Table 4.

### Identification of *Kerstersia gyiorum* using MALDI-TOF MS

Identification was carried out using MALDI-TOF MS as previously described (Schröttner et al., 2014; Schröttner et al., 2016). Pure bacterial cultures were applied to an MBT-Biotarget 96 plate (Bruker Daltonik GmbH, Bremen, Germany) using a sterile toothpick (Corning, Kaiserslautern, Germany). Once dried, formic acid (Merck, Darmstadt, Germany) was applied to release the proteins and improve binding of the subsequent matrix, α-cyano-4-hydroxycinnamic acid (HCCB) (Bruker Daltonik GmbH, Bremen, Germany). This treatment enhances ionization efficiency and improves the accuracy of the resulting mass spectra (Croxatto et al., 2012). Once dry, the sample was inserted into the MALDI Biotyper (Bruker Daltonik GmbH, Bremen, Germany). Analysis was performed using Flex Control Software 3.4 (Bruker Daltonik GmbH, Bremen, DE). The results were presented as a score ranging from 0 to 3 (Kostrzewa et al., 2019).

### Identification of *Kerstersia gyiorum* using results from sequencing of the 16S rRNA gene

In this study, PCR amplification was performed using the primer pair 27F (5′-AGAGTTTGATCMTGGCTCAG-3′)/1492R (5′-GGTTACCTTGTTACGACTT-3′), following a previously described protocol (Kopf et al., 2021). These primers cover the entire 16S rRNA gene and were chosen because they are commonly employed in phylogenetic studies (Johnson et al., 2019; Abellan-Schneyder et al., 2021). Subsequent PCR and sequencing were performed on two separate days to ensure the consistency of the results. In addition to the sequences from the clinical collection (*n* = 15), the type strain sequence was also analyzed.

The PCR products were then purified using Exonuclease I and Shrimp Alkaline Phosphatase, according to the manufacturer’s recommendations (New England BioLabs GmbH, Frankfurt, Germany). Sanger sequencing was then conducted by Microsynth Seqlab GmbH (Göttingen, Germany) under contract. Afterwards, the results were analyzed using BLASTN (Basic Local Alignment Search Tool) v. BLAST+ 2.17.0 algorithm (Abellan-Schneyder et al., 2021; Altschul et al., 1990; Camacho et al., 2009). The results are shown in Table 4. Quality control of the sequences was ensured by evaluating chromatograms in BioEdit and performing a plausibility check using MUSCLE alignment in MEGA 12 (Kumar et al., 2024; Hall, 1999). The proposed threshold of 98.65% was designated as the cutoff for species differentiation based on 16S rRNA gene sequencing (Kim et al., 2014). The forward and reverse sequences of the 16S rRNA genes were merged using MEGA 12 and aligned using MUSCLE (Edgar, 2004). The sequence of the type strain (LMG 5906^T^, DSM 16118^T^, CCUG 47000^T^) was obtained from LPSN (Freese et al., 2026). Subsequently, the sequences were aligned to the type strain.

## Results

### Characteristics of clinical data from the literature search

A total of 34 cases associated with *K. gyiorum* infection were identified (Table 1). As the data set did not include specific details regarding the age and sex of the patients in five cases, this information must be derived from the remaining publications. The patients’ ages ranged from 13 to 92 years, reflecting a broad demographic. The total number of patients included in the study was 13 females and 16 males. The distribution of cases by region is as follows: Europe (*n* = 3), the United States (*n* = 5), South America (*n* = 2), Asian countries, including Turkey (*n* = 16), the Arabian Peninsula (*n* = 1), and Africa (*n* = 3) (see Figure 1).

Most case reports are associated with ear disorders (*n* = 14) chronic wounds or ulcers (*n* = 5). Furthermore, cases of invasive infections, including bacteremia and sepsis (*n* = 1), local infections (*n* = 2), respiratory infections (*n* = 2), dysuria (*n* = 1), osteomyelitis (*n* = 1), and infections of unknown location (*n* = 1) have been reported. The available reports indicate the presence of alcohol and/or nicotine dependence (*n* = 5), poor hygienic living conditions (*n* = 1), and low awareness of illness (*n* = 2). One case report described a fatal outcome, but the others were successfully treated (Deutscher et al., 2014). Indwelling medical devices appear to be a risk factor for developing infection. Tracheostomies and urinary drainage procedures have been reported (Deutscher et al., 2014; Li et al., 2019; Ogawa et al., 2016; Sun et al., 2023). Furthermore, all patients had comorbidities. An exception appears to be patients with chronic suppurative otitis media (CSOM), as this primarily affects young, healthy patients.

In total, 3 cases have been reported in which monobacterial growth of *K. gyiorum* was detected. In other cases, relevant information is often missing, preventing a clear classification. Table 2 summarizes the identification methods for *K. gyiorum.* MALDI-TOF MS Biotyper (*n* = 18) (Bruker Daltonics GmbH, Bremen, Germany) and 16S rRNA gene sequencing (*n* = 25) were reported to be reliable identification methods. In contrast, VITEK2 (*n* = 14) and VITEK MS (*n* = 6) (probably due to limitations in the database) appeared less reliable. Whole-genome sequencing was performed in only 2 cases. In one instance, it was necessary because VITEK MS misidentified the isolate (Shen et al., 2025). No negative results were reported for identification with the MALDI-TOF MS Biotyper.

### Characteristics of the clinical collection

A total of 15 isolates were collected at University Hospital Dresden between December 2012 and July 2020, which were primarily identified as *K. gyiorum* (“clinical collection”). The youngest patient from whom strains were collected was 22 years old, and the oldest was 84. The average age was therefore 65 years. The patients included 9 women and 6 men. Samples were mainly taken from wound swabs (*n* = 9), followed by urine (*n* = 3), the ear canal (*n* = 2), and the throat (*n* = 1). Patients often had chronic leg ulcers, as well as urinary tract infections and middle ear infections. All strains were part of a polymicrobial spectrum. Depending on the collection site, the other bacteria were typical local pathogens (e.g., *Escherichia coli* in urine). Nevertheless, *Pseudomonas aeruginosa* was also isolated alongside typical bacteria.

### Results obtained from whole genome analysis of *Kerstersia gyiorum*

The *K. gyiorum* strains were identified using dDDH compared with the type strain 16618^T^. Apart from DSM 105837, all strains with dDDH values between 80.7 and 91.5% were reliably identified as *K. gyiorum* (see Table 4). As described before, these results fulfill the criteria for correct bacterial identification at the species level (Goris et al., 2007; Auch et al., 2010). Strain DSM 105837 revealed a value of 29.4% in reference to the type strain and is therefore well below the 70% cutoff for species identification using dDDH; it can be hypothesized that this is a bacterial species not yet described (Coenye et al., 1999) (see Table 4). Consequently, 14 of the 15 isolates from the “clinical collection” were identified at the species level as *K. gyiorum* using the dDDH. These 14 strains constituting this collection build the “reference collection” for the characterization of the species *K. gyiorum*. To ascertain potential relationships between the individual isolates, the genome sequences were initially analyzed using the Genome-to-Genome Distance Calculator (Wang et al., 2022). These analyses revealed that the strains DSM 108180 and DSM 109003, as well as DSM 108180 and DSM 110459, and DSM 109003 and DSM 110459, are completely identical at the genome level. This resulted in a DDH estimate of 100% for the first pair, DSM 108180 with DSM 109003. The GC difference is 0.0%, as are the distances. The comparison of DSM 108180 and DSM 110459 showed an average DDH estimate of 99.9%, a GC difference of 0.08%, and a maximum distance of 0.008. The final pair—DSM 109003 and DSM 110459—exhibited identical values. To provide further support for these results, analyses of the reference collection were conducted using both an ANI matrix and a pan-genome analysis. The thorough analysis of the pangenome using PanExplorer revealed that the *K. gyiorum* isolates consist of a substantial core genome (52.4%), an accessory genome (24.1%), and strain-specific genes (23.5%) (Dereeper et al., 2022) (Figure 2). To provide additional support for the analysis performed with PanExplorer, the numbers of genes in the core and accessory genome regions were determined using ROARY (Page et al., 2015; Galaxy, C, 2024). The results of this study are presented in Table 5. Conversely, the analysis of the ANI matrix yielded two distinct clusters (Figure 3). The ANI matrix in question was generated with JSpeciesWS, and the results were visualized with Morpheus. The initial, more compact cluster comprises isolates DSM 109001, DSM 109564, and DSM 110458. In contrast, a second, larger cluster was identified, consisting of isolates DSM 110459, DSM 108180, DSM 109003, DSM 109002, DSM 103762, DSM 108176, DSM 111617, DSM 111618, DSM 107564, DSM 107745, and DSM 111616.

**Figure 2:**
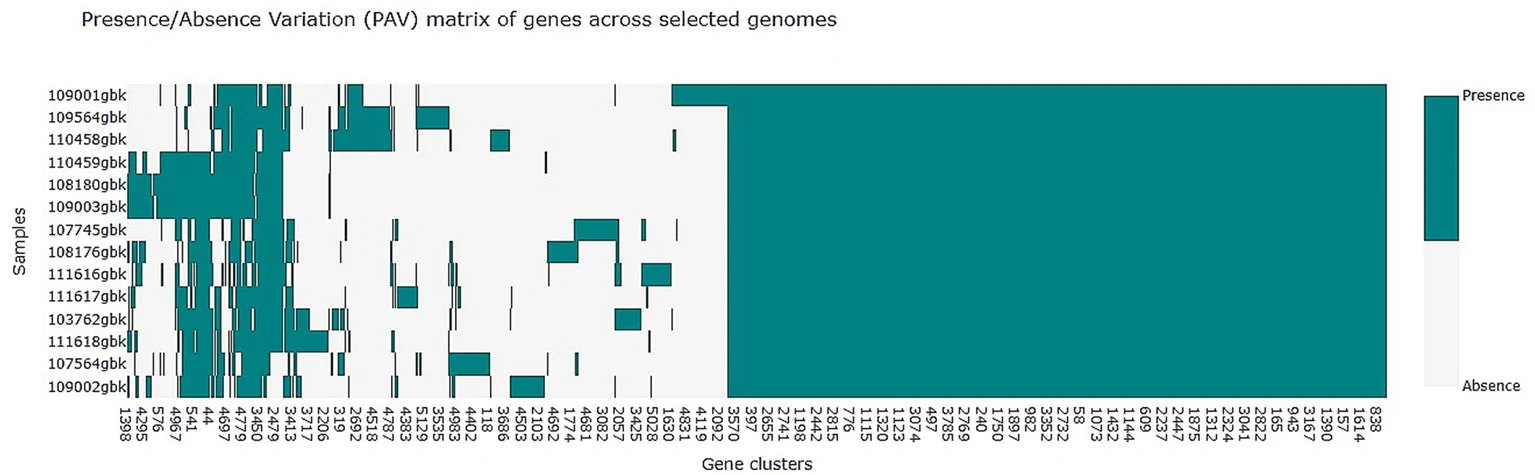
Presence/absence variation (PAV) matrix of genes of the reference collection. The Presence/Absence Variation Matrix for the genomes in the reference collection presented here was generated using PanExplorer v.2 via bio.tools. It shows that *K. gyiorum* possesses a very large pan-genome comprising about half of all available genomes. In contrast, the accessory genome and strain-specific genes make up the other half of the pan-genome. Creative Commons Attribution license (CC BY 4.0).

**Table 5:** Composition of the pangenome of the reference collection.

Genes	Number
Core genes	2663
Shell genes	1032
Cloud genes	1893
Total genes	5588

This table was derived from the pan-genome analyses performed using ROARY.

**Figure 3:**
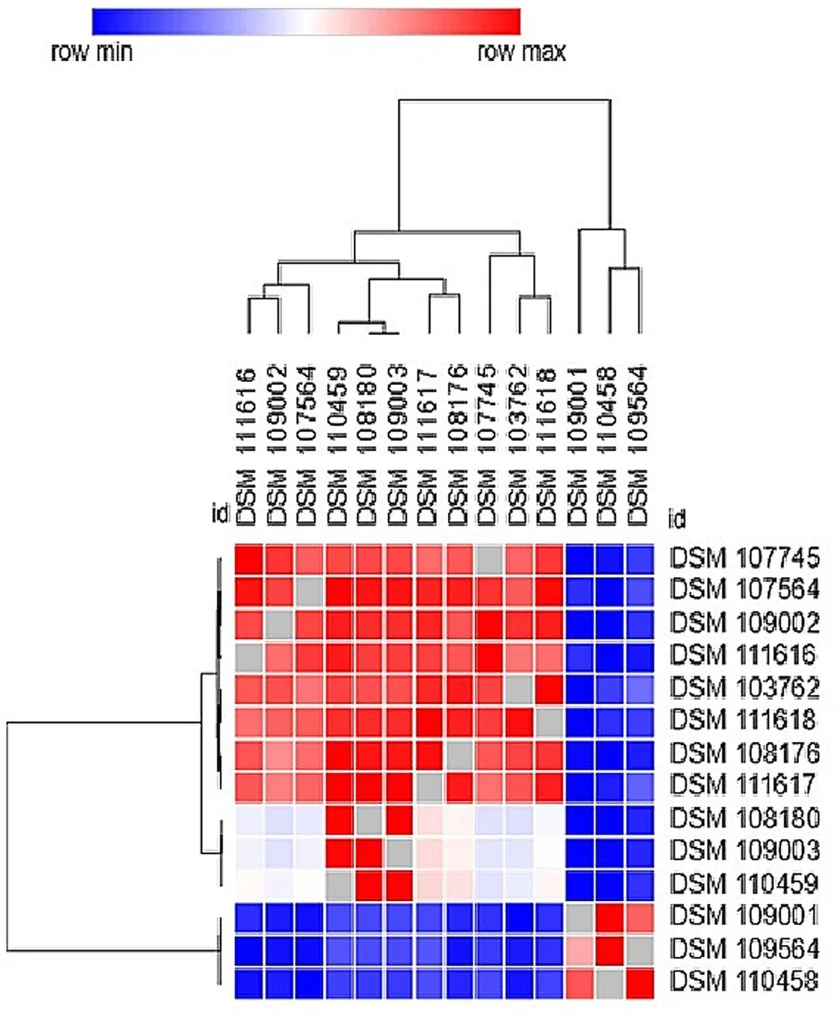
ANI matrix of genomes from the *Kerstersia gyiorum* reference collection. The ANI matrix in question was generated with JSpeciesWS, and the results were visualized with Morpheus. The analysis reveals two distinct clusters in the reference collection (for details, see the manuscript).

The Presence/Absence Variation Matrix for the genomes in the reference collection presented here was generated using PanExplorer. It shows that *K. gyiorum* possesses a very large pan-genome comprising about half of all available genomes. In contrast, the accessory genome and strain-specific genes make up the other half of the pan-genome (see Figure 2).

### VITEK 2

For evaluation purposes, the results are divided into quality-criteria percentages, with 93–95% indicating a “very good” identification and 96–99% indicating an “excellent” identification (Schröttner et al., 2016). Identification with VITEK 2 yielded four *Pandoraea* spp. results (DSM 105837, DSM 109002, DSM 109564, DSM 111616) with an average probability of 94%. Further results showed *Pseudomonas fluorescens* four times (DSM 103762, DSM 107564, DSM 110459, DSM 111618) and *Pseudomonas alcaligenes* once (DSM 108176). It should be noted, however, that no rating of the Advanced Expert System (AES) was given regarding the quality of the identification. The other seven strains could not be identified using VITEK 2, as shown in Table 4.

### MALDI TOF MS

A total of 14 strains from the clinical collection were identified as *K. gyiorum* by MALDI-TOF MS, with scores >2.0 (see Table 4). The exception was strain DSM 105837, which had an average score of 1.94 and was thus confirmed only at the genus level. According to the manufacturer’s guidelines, all values above 2.0 are considered reliable for identification at the genus and species levels. Thus, except for strain DSM 105837, all were identified as *K. gyiorum* at the species level.

### Sequencing of the 16S rRNA gene

The results of the bidirectionally sequenced PCR products were analyzed by matching to entries of the GenBank database of the National Center for Biotechnology Information (NCBI) (Washington, D.C., USA).^2^ The sequence identity was above 99.5% for *K. gyiorum* in all results. However, this was also the case for *Bordetella* spp. Interestingly, the strain DSM 105837 also differs from the other isolates analyzed using this method, as it has 100% sequence identity with *Alcaligenes* spp. (see Table 6). With values exceeding 98.7%, the criteria for correct bacterial species assignment were met in all sequencing runs (Altschul and Lipman, 1990). Furthermore, analysis of the sequences showed that *K. gyiorum* was the best match for all isolates except four. For these isolates DSM 109001, DSM 108180, DSM 107745 and DSM 109564, Bordetella ssp. was identified as the best match. Based on the results presented here, it is therefore not possible to differentiate between *Bordetella* spp., *Alcaligenes faecalis,* and *K. gyiorum* by sequencing the 16SrRNA gene. However, it should be noted that, upon examination of the actual sequence lengths of the 16S rRNA gene, it was not possible to map the entire gene, which spans approximately 1.5 kb (Table 4).

**Table 6:** Overview of MALDI-TOF MS scores for interpretation of the identification results (Bruker Daltonik GmbH, Bremen, Germany) (Kostrzewa et al., 2019).

Score	Description
2.00–3.00	High level of identification security
1.70–1.99	Low level of identification security
0.00–1.69	No reliable identification

## Discussion

Rare pathogens (such as *K. gyiorum*) are mostly opportunistic, and thus infections caused by these bacteria are usually associated with immunosuppression or chronic disease. For instance, an infection with *Myroides odoratus* was detected in an HIV-positive patient (Manfredi et al., 1999). Another example is the case of a patient with systemic lupus erythematosus who developed an endocarditis caused by *Roseomonas mucosa* (Shao et al., 2019). A similar association is observed for *K. gyiorum*, as case studies of *K. gyiorum* infections often occur alongside chronic inflammatory conditions (Pence et al., 2013; Almuzara et al., 2012; Mwalutende et al., 2014; Uysal et al., 2015; Kitagawa et al., 2021). These include chronic wounds of the lower extremities and, in particular, chronic ear diseases. Open entry points due to a tracheostomy or nephrostomy appear to be an additional risk factor (Li et al., 2019; Ogawa et al., 2016). Furthermore, it is interesting to note that alcohol and nicotine abuse, as well as poor hygiene and patient non-compliance, were explicitly mentioned in some reports (Almuzara et al., 2012; Baran et al., 2017; Bostwick et al., 2015; Kitagawa et al., 2021). This finding could therefore indicate that *K. gyiorum* infections could be preventable through targeted hygiene measures. For example, changing drainage tubes improved the collection situation in one case (Ogawa et al., 2016). However, because only one known case is associated with malignant comorbidity, this association remains questionable (Ogawa et al., 2016). Nonetheless, the available data are very limited. For this reason, appropriate methods should be used to reliably identify *K. gyiorum,* and an accompanying prospective scientific observation should be conducted to provide greater clarity on potential risk factors for future *K. gyiorum* infection.

As previously mentioned, *K. gyiorum* was detected alongside other bacteria in most published cases. In this context, Mwalutende et al. (2014) hypothesize that *K. gyiorum* primarily occurs in patients with chronic mixed infections. Additionally, Ogawa et al. (2016) assume that *K. gyiorum* thrives in environments conducive to polymicrobial infections, such as those associated with chronic diseases and poor hygiene. This hypothesis is based on *K. gyiorum’s* close relationship with the genera *Achromobacter* and the family *Alcaligenaceae*, which are often isolated as part of a polymicrobial spectrum in immunocompromised patients, especially in those with malignant diseases (Ogawa et al., 2016; Coenye et al., 1999). However, Li et al. (2019) point out that there is currently insufficient data available to substantiate these assumptions scientifically.

By contrast, only three cases have been published to date in which *K. gyiorum* was the sole bacterium identified (Özcan, 2018; Bostwick et al., 2015). These cases will be described in more detail below, as monobacterial growth suggests that *K. gyiorum* triggered the disease. In the first case, bacteremia with subsequent sepsis was reported (Bostwick et al., 2015). *K. gyiorum* was detected in two sets of blood cultures, but no information was provided regarding whether the samples were drawn from a peripheral or central line. The site of entry was probably the large ulcers on the lower extremities (Bostwick et al., 2015). The other two cases were published by Özcan (2018). One case is described as presenting with ear discharge, perforation of the left tympanic membrane, and cholesteatoma (no previous illnesses, non-smoker). The second patient has had ear discharge since childhood and is a smoker (Özcan, 2018). Although *K. gyiorum* was found to be resistant to ciprofloxacin in the first case, both patients were successfully treated with ciprofloxacin ear drops and ceftriaxone. The authors explain this by noting that the drops create higher antibiotic concentrations directly at the site of infection, thereby achieving an antimicrobial effect. The authors also suggested that *K. gyiorum* could be a potential causative agent of chronic suppurative otitis media (CSOM) (Özcan, 2018). Furthermore, there is speculation as to whether *K. pneumoniae* colonizes the respiratory tract or causes respiratory infections (Almuzara et al., 2012; Deutscher et al., 2014; Li et al., 2019).

Examining the Dresden cases reveals that most patients in this group were female and that the average age of 65 years was significantly higher than that reported in published studies (see Table 3). Furthermore, despite ear canal samples being mentioned frequently in the literature (*n* = 11), *K. gyiorum* was sucessully detected in only two ear canal swabs (Pence et al., 2013; Mwalutende et al., 2014; Özcan, 2018; Uysal et al., 2015; Borsa et al., 2017; Kim et al., 2018). These two patients from Dresden with ear swabs are, on average, 36 years old. The average age of patients in the published literature is 38 years (see Table 1). In contrast, the average age of nine patients with a wound infection in the ‘Dresden collection’ is 70 years (see Table 3). However, only four patients with wound infection were reported in the literature. The average age of these patients was 50 years (see Table 1) (Pence et al., 2013; Baran et al., 2017; Kitagawa et al., 2021; Lan et al., 2017). These data therefore suggest that ear infections caused by *K. gyiorum* affect younger people more frequently, while older patients are more likely to suffer from wound infections. Nevertheless, further prospective observation of clinically evident infections associated with *K. gyiorum* is required.

As previously mentioned, identifying *K. gyiorum* reliably in routine diagnostics is key to further epidemiological and laboratory-based investigations. For this reason, the results of the comparison of routine methods will now be discussed and evaluated. The most important prerequisite for delivering meaningful results in methodical comparative studies is the use of a reliable reference method. In this study, we applied dDDH because next-generation sequencing (NGS)-based methods of genomic analysis are currently considered the molecular gold standard for species identification and are particularly well suited for taxonomic comparisons (Richter and Rossello-Mora, 2009; Chun et al., 2018). NGS was carried out using “PacBio,” a so-called “third-generation method,” representing ‘long-read technology’. This means that an entire closed genome can be sequenced in a single run (Altschul and Lipman, 1990; Goodwin et al., 2016; Deurenberg et al., 2017). Furthermore, third-generation methods (PacBio and Oxford Nanopore) offer the possibility of detecting methylation and other chemical modifications that can play important roles in regulating bacterial biology or pathogen virulence (Auch et al., 2010; Korlach and Turner, 2012; Loman and Pallen, 2015). However, the main disadvantage is that this technology cannot yet be integrated into routine diagnostics. In addition to the necessary infrastructure, the technology must be developed to the point where it can be used in routine diagnostics. However, this is not yet possible due to high costs, a lack of trained specialists, and the time and financial resources required (Behjati and Tarpey, 2013; Ardui et al., 2018). Nevertheless, the advantages of this technology are clear: NGS-based analyses yield highly precise genomic data due to a very low error rate and short turnaround time (Auch et al., 2010; Meier-Kolthoff et al., 2013; Meier-Kolthoff, 2019). This is reflected in the genome data results of the study collection presented here: 14 genomes of the reference collection and the type strain were clearly assigned to the species *K. gyiorum* using dDDH. A striking feature of the dDDH analysis was that three genome pairs matched 100% (DSM 108180 and DSM 109003; DSM 108180 and DSM 110459; or DSM 109003 and DSM 110459), indicating a common origin of the strains. However, because the strains were taken from different patients or at different times, it is necessary to assess whether the infections are connected or share a common origin. The results presented in this manuscript suggest that DSM 108180 and DSM 109003 may be of the same clonal origin. The other two comparisons show that the isolates in the different patients were not the same or had changed slightly. However, since dDDH is primarily used to address taxonomic questions, we additionally included an ANI-based analysis to verify the results. ANI is particularly well-suited for verifying phylogenetic clustering and identifying closely related isolates (Goris et al., 2007). Interestingly, the ANI analysis yielded results that differed from those obtained by dDDH (see Figure 3). It showed a clear distinction between two clusters (DSM 1090001, DSM 109564, DSM 110458 and DSM 110459, DSM 108180, DSM 109003, DSM 109002, DSM 103762, DSM 108176, DSM 108176, DSM 111617, DSM 111618, DSM 107564, DSM 107745, DSM 111616) within the pan-genome of the reference collection (see Figure 3). To characterize the underlying gene repertoire, an additional PAV analysis was performed, confirming the ANI analysis results. The analysis revealed that the smaller cluster (DSM 1090001, DSM 109564 and DSM 110458) differs in its accessory genome from the other isolates (e.g., gene clusters 44 and 2092) (see Figure 2). Consequently, in this study, we identified a discrepancy between the ANI-based analysis and the dDDH results. Such a discrepancy between ANI and dDDH values had already been reported by Li et al. (2026). During a taxonomic analysis of the genus *Sphingobacterium*, the authors noted that although the ANI values for all isolates were above 95%—and thus above the cut-offs defined for a species—the dDDH values for individual isolates were below 70% (Li et al., 2026). This finding, however, contradicts the species identified by ANI (Li et al., 2026). Therefore, since the results of the dDDH analysis could not be validated by subsequent analyses in the present study, the following procedure is recommended for future comparative genomic studies. Initially, the relationships among the strains are determined through an ANI-based comparison. After this, confirmation is to be made via a PAV analysis based on the evaluation of the pan-genome. If ANI values fall below the 95% cutoff—and thus outside the defined species boundaries—species confirmation is to be performed using dDDH. Moreover, the pan-genome analysis revealed that *K. gyiorum* possesses a substantial core genome. This observation suggests that *K. gyiorum* occupies a stable ecological niche and is subject to only minimal selective pressure. This finding could be useful in the search for the species’ natural habitat (Brockhurst et al., 2019).

In contrast, identification using the VITEK 2 system is based on analyzing the biochemical and metabolic properties of bacteria (Altschul and Lipman, 1990; Funke et al., 1998). However, investigations into the identification of rare bacterial pathogens revealed limitations of the VITEK 2 system in previous studies. For instance, Rudolph et al. (2019) demonstrated that *R. mucosa* (confirmed using ANI) was misidentified as *Roseomonas gilardii* using the VITEK 2 system. Additionally, Kopf et al. (2021) showed that *Wohlfarthiimonas chitiniclastica* was misidentified as *Acinetobacter lwoffii*. In this study, dDDH was also used as the reference method (Kopf et al., 2021). The literature also points out difficulties in identifying *K. gyiorum* using VITEK 2, with negative results in each case (Pence et al., 2013; Mwalutende et al., 2014; Kim et al., 2018). These inadequate results are probably due to the characteristics of *K. gyiorum* not being stored in the VITEK 2 database (Almuzara et al., 2012; Bostwick et al., 2015). Another hypothesis is that false results could be due to biochemical characteristics similar to those of *Acinetobacter* spp. (Baran et al., 2017; Mwalutende et al., 2014). This observation is also reflected in the results presented in this manuscript: in 7 cases, identifications were not possible, and in 8 cases, wrong (*Pseudomonas n* = 4; *Pandorea* spp. *n* = 3 and *Pseudomonas alcaligines n* = 1) (see Table 6). Therefore, the hypotheses of Mwalutende et al. (2014) and Baran et al. (2017) that some strains were, in fact, *Acinetobacter* spp. can be refuted, as no strain was identified as such. In summary, VITEK 2 is not a suitable method for identifying *K. gyiorum* (Almuzara et al., 2012; Baran et al., 2017; Özcan, 2018; Uysal et al., 2015).

Another method used in routine diagnostics to identify bacterial species is sequencing of the 16S rRNA gene. Several authors have primarily used this method to identify *K. gyiorum* (Coenye et al., 2003; Almuzara et al., 2012; Bostwick et al., 2015; Kitagawa et al., 2021). However, in other studies, this method has also been used to confirm an existing identification result (Pence et al., 2013; Li et al., 2019; Vela et al., 2017; Kim et al., 2018). A review of the available literature shows that *K. gyiorum* was identified with 99–100% agreement with the ‘NCBI GeneBank and EZ Taxon database’ (*n* = 9). *K. similis* was described as the next best match four times with 98–99.6% similarity. Interestingly, *Bordetella* spp., *Alcaligenes* spp., and *Achromobacter* spp., which also belong to the Alcaligenaceae family, also showed matches in the range of 95–99% (Pence et al., 2013; Bostwick et al., 2015; Ogawa et al., 2016). Nevertheless, to underpin the reliability of the method, Bostwick et al. explicitly point out that despite being closely related to *A. faecalis* and despite the availability of relevant database data (Biolog Gen III, version 2.7.1), *K. gyiorum* was not identified as *A. faecalis* (Bostwick et al., 2015; Li et al., 2019). However, the results of the 16S rRNA sequencing conducted in this study differ significantly from those reported in the literature. All the strains contained within the reference collection yielded comparable results for both *K. gyiourum* and *Bordetella* spp. One possible reason for this is the close phylogenetic relationship between *K. gyiorum* and these genera. As previously mentioned, *Bordetella* spp., *Alcaligenes* spp. and *K. gyiorum* are all members of the Alcaligenaceae family. This may also explain the sequence similarity of the 16S rRNA gene between *K. gyiorum* and other genera. Coenye et al. (1999, 2003) already addressed this issue of sequence similarities in their original description. They demonstrated that the 16S rRNA gene sequences of *K. gyiorum* are most similar to those of the genera *Bordetella* (96.4%), *Achromobacter* (95.1%), *Alcaligenes* (94.8%), and *Pigmentiphaga* (93.5%). These data clearly illustrate that sequencing errors or incorrect database entries may render identification based on the 16S rRNA gene unreliable. It should also be noted that the database used to analyze the sequences in this study is not curated. Furthermore, the full 16S rRNA gene sequence was not recorded for any of the isolates. While the best match was consistently *K. gyiourum*, misinterpretations in subsequent matches due to insufficient sequence accuracy or technical issues cannot be ruled out. In contrast, the reference method (dDDH) correctly identified all strains except one as *K. gyiorum*. This one strain (DSM 105837) also received a lower rating in MALDI-TOF MS (see Table 6), suggesting it may be a not yet described species within the underlying genus. Consequently, MALDI-TOF MS results appear to be particularly important for identifying bacteria at the genus level. This assertion is further substantiated by the findings of previous studies conducted by our research group. Rudolph et al. (2019), for instance, showed that *R. mucosa* scores ˃ 2 were consistent with the reference method, “Average Nucleotide Identity” (ANI). However, for one isolate, the result was below 2.0 and was therefore identified at the genus level. In this case, the both ANI and dDDH results indicated the species *R. gilardii*. Furthermore, Kopf et al. (2021) clearly demonstrated that a MALDI-TOF MS identification result of *W. chitiniclastica* with a score greater than 2.0 directly correlates with the dDDH results. Based on these results, it is highly likely that MALDI-TOF MS is also the best method for identifying *K. gyiorum* in routine diagnostics. However, there have been reports of incorrect *K. gyiorum* strain identifications when using VITEK MS (bioMérieux, Nürtingen, Deutschland), another MALDI-TOF MS platform (Bostwick et al., 2015; Kitagawa et al., 2021). The extant literature has identified the absence of corresponding reference spectra as a potential contributing factor to this phenomenon (Holmes et al., 2021; Pence et al., 2013). However, a study demonstrated the suitability of VITEK MS for the identification of *K. gyiorum*, as the authors entered spectra into the scientific database (RUO) and identified the isolates in a follow-up analysis (Zhang et al., 2025). In this study, *K. gyiorum* was also confirmed using alternative methods. In addition, a report has been published on the successful identification of *K. gyiorum* using VITEK MS from 2024 (AlSunbul et al., 2024). This suggests that the reference spectra may already be available. However, it is important to acknowledge the absence of systematic studies that have examined the identification of defined *K. gyiorum* isolates using VITEK MS. The reference collection presented in this manuscript could therefore be suitable for this purpose as well.

Finally, the use of MALDI-TOF MS for the identification of *K. gyiorum* will be discussed. One major advantage of MALDI-TOF MS is that it is now a well-established and widely used method in routine microbial diagnostics (Schröttner et al., 2016; Croxatto et al., 2012). It is therefore also an excellent screening method for identifying rare bacterial pathogens. In this study, MALDI-TOF MS successfully identified *K. gyiorum* in 14 of 15 strains of the clinical collection, achieving values above 2.0 at the species level. These results were consistent with identification by dDDH (our reference method) (see Table 6). For this reason, MALDI-TOF MS seems to be a reliable method for identifying *K. gyiorum*. These observations are also consistent with previously published data (Pence et al., 2013; Li et al., 2019; Kim et al., 2018).

Taken together, it is evident that the VITEK 2 system is not suitable for identifying *K. gyiorum*. Contrary to reports in the literature, 16S rRNA analysis was also found unsuitable in this study. However, MALDI-TOF MS and dDDH appear to be robust identification methods, with MALDI-TOF MS being the most widely used in routine diagnostics. These methods produce consistent results, which is why they should be favored to allow correct identification. Furthermore, it is also noteworthy that *K. gyiorum* is typically observed as part of a polymicrobial spectrum. Consequently, it can be challenging to ascertain whether this species is the primary causative agent of the infection or if it is merely a result of colonization. Conversely, in the latter scenario, no pathogenic significance can be attributed to this species. For this reason, infections with evidence of a single bacterial species should continue to be accorded particular significance in the assessment of pathogenicity in future investigations.

## Conclusion

Since the initial description of *K. gyiorum* in 2003, an increasing number of clinical case reports of infections caused by *K. gyiorum* have been published. This manuscript represents an overview of all published cases to date, as well as the status of risk factors and available genomic data. The 15 strains of the ‘Dresden Collection’ (“clinical collection”) were collected over 8 years and analyzed for various characteristics. Chronic wounds and middle ear infections are identified as risk factors for acquiring an infection. However, no comparative statement can be made regarding the poor hygiene and living conditions described in isolated cases due to a lack of references.

Furthermore, conventional methods were found unsuitable for identifying bacterial species. Reliable and precise diagnostics can be achieved using MALDI-TOF MS and NGS-based dDDH. In contrast, sequencing the 16S rRNA gene did not lead to suitable results in the present study, and a reliable differentiation from members of the *Alcaligenaceae* could not be achieved. This should be considered in the future for patients with long-term chronic wounds and poor wound conditions, as well as long-term ear infections and otorrhoea, to treat the patient in a targeted manner. It is noteworthy that this research has culminated in the establishment of a meticulously delineated reference collection of 14 strains, which has been made accessible to the scientific community through the “Open Collection” of the DSMZ. This signifies a substantial contribution to “Open Science.”

## Data Availability

The datasets presented in this study can be found in online repositories. The names of the repository/repositories and accession number(s) can be found at: https://www.ncbi.nlm.nih.gov/, JBXEKT000000000, JBXEKS000000000, JBXEKR000000000, JBXEKQ000000000, JBXEKP000000000, JBXEKO000000000, JBXEKN000000000, JBXEKM000000000, JBXEKL000000000, JBXEKK000000000, JBXEKJ000000000, JBXEKI000000000, JBXEKH000000000, JBXEKG000000000, JBXEKF000000000.

## Footnotes

1
https://tygs.dsmz.de

2
https://blast.ncbi.nlm.nih.gov/Blast.cgi
