# Supplementary material for: Comparison of three routine methods for the identification of Kerstersia gyiorum from routine diagnostics and review of the literature
Source: Front Microbiol. 2026 Oct 5;17:1901541. doi: 10.3389/fmicb.2026.1901541 (PMC13679296; doi:10.3389/fmicb.2026.1901541)
Supplement: Supplementary file 1 [file Data_sheet_1.pdf]

	103762.fsa	107564.fsa	107745.fsa	108176.fsa	108180.fsa	109001.fsa	109002.fsa	109003.fsa	109564.fsa	110458.fsa	110459.fsa	111616.fsa	111617.fsa	111618.fsa
103762.fsa	*	98.52	98.67	98.78	98.63	97.37	98.63	98.63	97.69	97.55	98.67	98.61	98.75	98.86
107564.fsa	98.56	*	98.67	98.72	98.75	97.56	98.63	98.75	97.64	97.44	98.80	98.75	98.71	98.78
107745.fsa	98.77	98.78	*	98.80	98.84	97.73	98.89	98.84	97.88	97.77	98.84	99.02	98.75	98.88
108176.fsa	98.87	98.75	98.81	*	99.00	97.62	98.65	99.00	97.68	97.60	99.06	98.82	99.02	98.91
108180.fsa	98.70	98.75	98.71	98.90	*	97.69	98.71	100.00	97.85	97.69	99.98	98.77	98.99	98.81
109001.fsa	97.49	97.54	97.64	97.61	97.71	*	97.57	97.71	98.78	99.08	97.72	97.63	97.71	97.64
109002.fsa	98.59	98.54	98.71	98.50	98.60	97.44	*	98.60	97.56	97.44	98.64	98.53	98.62	98.64
109003.fsa	98.72	98.77	98.72	98.91	100.00	97.68	98.71	*	97.85	97.70	99.97	98.78	99.01	98.83
109564.fsa	97.60	97.55	97.71	97.57	97.77	98.71	97.57	97.77	*	99.31	97.80	97.51	97.81	97.68
110458.fsa	97.51	97.44	97.72	97.58	97.66	99.08	97.55	97.66	99.41	*	97.68	97.50	97.73	97.69
110459.fsa	98.76	98.86	98.72	98.98	100.00	97.70	98.79	100.00	97.91	97.69	*	98.87	99.03	98.89
111616.fsa	98.65	98.82	98.98	98.74	98.81	97.67	98.67	98.81	97.60	97.54	98.91	*	98.80	98.67
111617.fsa	98.84	98.80	98.76	99.00	99.07	97.67	98.72	99.07	97.94	97.75	99.07	98.83	*	98.99
111618.fsa	98.89	98.68	98.77	98.84	98.81	97.59	98.67	98.81	97.75	97.71	98.8	98.63	98.92	*
